# Amitriptyline Pharmacokinetics in Experimental Spinal Cord Injury in the Rabbit

**DOI:** 10.4103/0250-474X.49122

**Published:** 2008

**Authors:** H. Reihanikermani, M. Ansari, A. Soltani, M. S. Meymandi

**Affiliations:** 1 Department of Neurosurgery, Bahonar Hospital and Neuroscience Research Center, Kerman Medical Sciences University, Kerman, Iran; 2 Neuroscience Research Centre, Kerman Medical Sciences University, Kerman, Iran

**Keywords:** Amitriptyline, spinal cord injury, pharmacokinetic

## Abstract

Previous studies have demonstrated that pharmacokinetic behavior of several drugs such as paracetamol, theophylline, and aminoglycosides are significantly altered in spinal cord injured patients. No pharmacokinetic study of amitriptyline has been performed in patients and experimental models of spinal cord injury. Pharmacokinetic parameters of amitriptyline in orally treated rabbits subjected to laminectomy and spinal cord injury compared with those underwent laminectomy alone. Among twenty four male rabbits were included in this study, nine of them subjected to spinal cord injury at the 8^th^ thoracic level by knife severance method and six rabbits underwent laminectomy alone (sham group) and nine rabbits treated as control. All received a single oral dose of amitriptyline (20 mg/kg) 24 h after injury. Blood sampling were done at predetermined times to 36 h after drug administration. Amitriptyline concentration in serum samples was determined by high-performance liquid chromatography. Pharmacokinetic parameters including maximum concentration (C_max_), time to reach maximum concentration (T_max_), half life, and the area under the curve to last detectable concentration time point (AUC_0-t_) were directly determined from the concentration-time curve. Maximum concentration was observed at 6.5 h after administration in sham group with a concentration of 439.6 ng/ml, whereas in SCI group T_max_ was at 2.7 h with a concentration of 2763.9 ng/ml. In control group it was 3.3 h and 396 ng/ml, respectively. In SCI group, AUC was 9465.6 ng.h/ml and half life was 6 h and for control group it was 2817.4 ng.h/ml and 6.4 h, respectively. Statistical analysis of data showed that SCI didn't induce significant changes in amitriptyline pharmacokinetic parameters.

Morbidity and mortality rate after both human and experimental spinal cord injury (SCI) are high. Life threatening complications such as pneumonia, urinary tract infections and infected pressure sores are commonplace, mainly at the early stages of the lesion, despite the use of standard pharmacological treatment^1^–^3^. It is possible that such therapeutic failures are due, at least in part, to unsuitable dosing strategies which don't consider pharmacokinetic alteration in this patient population^4^. SCI may change the kinetics of drug absorption, distribution and elimination^5^. About 66% of all SCI patients reported some type of pain with the most common variety of pain consisted of a sensation usually described as burning in body parts below the level of injury^6^. There are evidences that pharmacokinetic behavior of several drugs^7^ such as paracetamol^8^, theophylline^9^, and aminoglycosides^10^ are significantly altered in SCI patients, but, to our knowledge, no pharmacokinetic study of amitriptyline has been performed in patients and experimental models during the acute phase of SCI. Therefore, we considered it of interest to study whether pharmacokinetics of amitriptyline is altered by spinal cord injury. Clinical reports on drug kinetics in spinal cord injury are often anecdotal, because it is extremely difficult to perform systematic pharmacokinetic studies in SCI patients. This difficulty is due to the important of inter-individual variability in injury extent and location^11^. Therefore the use of experimental models appears to be a suitable strategy for understanding the pharmacokinetic alteration due to SCI.

Amitryptyline was obtained from Sobhan Pharmaceutical Co. (Iran). Amitriptyline oral solution (20 mg/ml) was made in our laboratory. Rabbits (2500-3000 g) were used. Twelve hours before drug administration, food was with-held, but animals had free access to water. The study was approved by the local Animal Care Committee.

Animals were anesthetized by intraperitoneal injection of ketamine HCl (75 mg/kg) and thiopental sodium (50 mg/kg). Under aseptic conditions, laminectomy was performed at the T8 level; the spinal cord was completely severed by clean transversal cut by a scalpel^12^. Then aponeurotic plane and the skin were separately sutured with 5-0 nylon thread. Postsurgical care was performed as described by Guízar-Sahagún *et al*.^13^.

A single dose 20 mg/kg of amitriptyline as solution 20 mg/ml was given by gavages through a nasogastric tube. Blood samples (2 ml) were drawn at 0, 1, 2, 3, 4, 6, 8, 10, 12, 24 and 36 h after drug administration. The total blood volume extracted did not exceed 30 ml. Amitriptyline concentration in serum samples was determined by high-performance liquid chromatography, as described by López *et al*.^14^ The HPLC conditions were as follows: HPLC pump Knauer, K-10001; Detector, UV Knauer 2600, 250 nm; Autosampler, Knauer; Solvent organizer, Knauer K1500; Column, Knauer ODS 250×4.6 mm; Mobile phase (methanol 55%, buffer 37%, acetonitrite 8%), flow rate, 1.5 ml/min; internal standard, diltiazim 1 μg/ml. All chromatographic separations were performed at room temperature.

Individual serum concentration against time curves were constructed, and the maximum concentration (C_max_), as well as the time to reach the maximum concentration (T_max_), were directly determined from these plots. Half-life was estimated by linear regression of the terminal concentration decay phase plotted in semi-logarithmic coordinates. The area under the concentration-time curve (AUC) to the last concentration-time point was determined by the trapezoidal rule and extrapolated to infinity by dividing the last detectable concentration by the terminal slope. Absorption rate constant was estimated by residual method.

Three groups of rabbit were studied. Nine animals subjected to SCI and six animals were subjected only to laminectomy and served as a sham group and nine Rabbits studied as control group. Oral amitriptyline by gastric gavages was given 24 h after the surgical procedure. Comparisons between control, SCI and sham animals were performed by student's t test for unpaired data. Also 90% confidence interval was used to determine the differences between mean pharmacokinetic parameters in two groups. Differences were considered to reach statistical significance when p <0.05.

All the animals studied exhibited locomotor activity before the initiation of the study. After surgical procedure, injured rabbits showed complete flaccid paraplegia and five of them had watery stool after 12 h, whereas sham-injured animals exhibited normal walk after recovery from anesthesia. Serum amitriptyline concentrations observed at various times with a single oral dose of 20 mg/kg administered after SCI and under control conditions are shown in fig. 1. This figure indicates that C_max_ was observed at 3.3 h after administration in control group with a concentration of 396.0 ng/ml, whereas C_max_ in SCI group was appeared at 2.7 h with a concentration of 2763.9 ng/ml. These pharmacokinetic parameters are higher and variable in SCI group than control group. Pharmacokinetic parameters are shown in Table 1.

**Fig. 1:**
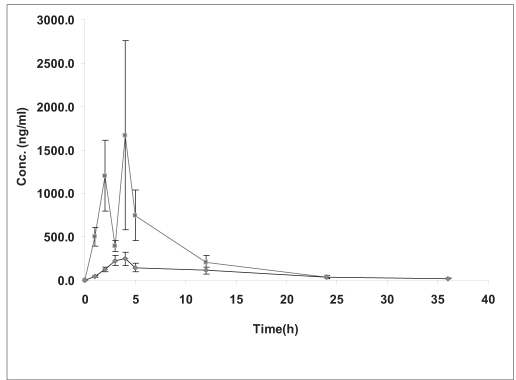
Concentration-time profile of amitriptyline in rabbits The profile obtained from administration of amitriptyline to (–▪–) SCI and (–•–) control groups of rabbits. Each point represents mean±SEM

**TABLE 1:** STATISTICAL COMPARISON OF THE MEAN VALUES OF PHARMACOKINETIC PARAMETERS

	Control Mean±SD (n=9)	SCI Mean±SD (n=9)	Sham Mean±SD (n=4)	t Test (Sham to Control)	t Test (Sham to SCI)
AUC_0-t_(ng.h/ml)	2654.3±1972.7	8812.0±5945.2	4594.8±4196.8	0.2	0.1
AUCI (ng. h/ml)	163.2±180.6	653.7±1251.2	165.5±55.1	0.2	0.3
AUC(ng.h/ml)	2817.4±2041.4	9465.6±6067.5	4760.3±4163.2	0.2	0.1
C_max_ (ng/ml)	396.0±183.8	2763.9±2876.9	439.6±260.6	0.2	0.1
t_max_ (h)	3.3±1.0	2.7±1.5	6.5±3.7	0.0	0.0
t_½_ (h)	6.4±3.3	6.0±5.5	7.7±1.7	0.5	0.1

Statistical comparison of the mean values of pharmacokinetic parameters of amitriptyline in different groups of rabbits including control, spinal cord injured, and sham group

The results showed that pharmacokinetic parameters calculated in this study was greater in SCI group compared to control group, despite this, t-student test between parameters has not shown any statistically significant difference between two groups.

It is known that SCI results in physiological disturbances which alter the pharmacokinetics of several therapeutic agents. However, drug treatment in these types of patients is still performed on an empirical basis, since no rational strategy has yet been provided. It is hence necessary to characterize the pharmacokinetic alterations induced by SCI in order to adequately design dosage regimens. Since it is difficult to perform systematic studies in patients, we have undertaken the characterization of SCI induced pharmacokinetic alteration using a well-described experimental model, i.e. spinal cord severance by knife method in the rabbit. Such characterization, however, is highly difficult to perform in human subjects, give the high inter-individual variability observed in clinical situation. Therefore pharmacokinetic studies using experimental model of SCI appear to be the most suitable strategy^4^. Amitriptyline was studied because it is widely used for treatment of pain in SCI patients^15^, and some evidence suggest that one of the most effective agent in treatment of neuropathic pain due to SCI is amitriptyline. The results of present study showed that SCI at the level T8 did not produce any significant alteration in amitriptyline pharmacokinetic parameters, although there was a trend toward increase in C_max_ and AUC and decrease in T_max_ and half life. Lara *et al.* found that SCI at T8 level lead to reduction in C_max_ and AUC of salicylic acid after oral Aspirin administration in rat, suggesting that SCI decreased the rate of aspirin absorption^11^,^16^. Lopez *et al*. had shown SCI at the level T8 significantly reduced paracetamol C_max_ and AUC after oral administration^4^. Since amitriptyline is only orally administered, it seems that any change in motility or secretion of GI tract induced by SCI could affect the rate and extent of absorption of it. In the present study, we observed that in contrast to some more above mentioned water soluble drugs, not only SCI did not decrease C_max_ and AUC of amitriptyline but it is increased to some extent these parameters, although this increase is not statistically significant. There is evidence that gastric emptying is impaired by SCI^9^,^17^, likely by a stimulation of nitric oxide release resulting in an inhibition of gastrointestinal motility^18^. It has been reported that SCI result in construction of several vascular territories including gastrointestinal wall^11^,^19^, therefore, other mechanisms cannot be discarded with the information available at present. The results of our study showed that half life of amitriptyline decreased in SCI, which did not reach statistical significant. A prolonged half life in experimental animals 24 h after SCI at the T8 level has also been reported for cyclosporine and diclofenac^20^,^21^. These reports suggest that SCI results not only in alterations in drug absorption, but also in drug elimination. The mechanisms in the impairment of drug elimination by SCI remain unclear^20^.
